# Single-session endoscopic ultrasound-directed transgastric endoscopy for treatment of upper gastrointestinal bleeding after Roux-en-Y gastric bypass

**DOI:** 10.1055/a-2197-9404

**Published:** 2023-11-21

**Authors:** Jordan Burlen, Albert Manudhane, Luke Roberts, Anna Cecilia Amaral, Georgios I. Papachristou, Samuel Han

**Affiliations:** 1 12306 Gastroenterology and Hepatology, The Ohio State University Wexner Medical Center, Columbus, United States; 2 12306 Gastroenterology, Hepatology, and Nutrition, The Ohio State University Wexner Medical Center, Columbus, United States; 3 12306 Division of Gastroenterology, Hepatology and Nutrition, The Ohio State University Wexner Medical Center, Columbus, United States

Endoscopic ultrasound (EUS)-directed transgastric endoscopic retrograde cholangiopancreatography (ERCP) – also known as EDGE – for the purpose of accessing the duodenum to perform EUS or ERCP has been well described in patients who have undergone Roux-en-Y gastric bypass (RYGB) 1 . Few reports, however, have described the use of EDGE to manage upper gastrointestinal bleeding in patients with RYGB anatomy 2 .

A 48-year-old woman (Jehovah’s Witness) with a history of RYGB presented with melena and a hemoglobin level of 9.0 g/dL. An upper endoscopy revealed a healthy appearing gastrojejunal anastomosis and no source of bleeding. The hemoglobin level dropped further to 5.0 g/dL and the patient had persistent melena. A computed tomography angiography was concerning for hemorrhagic fluid in the descending duodenum ( Fig. 1 ). After discussing possible treatment options with the patient, including balloon-assisted enteroscopy vs. single-session EDGE, the patient opted for the latter.

**Fig. 1:**
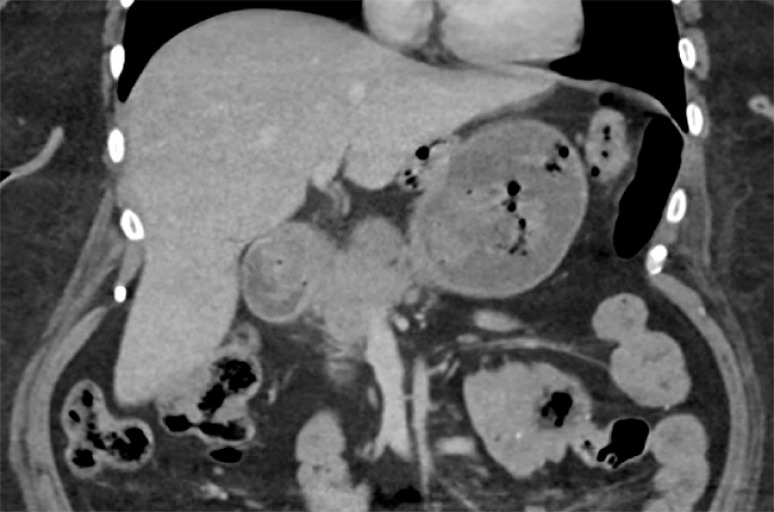
Computed tomography demonstrating high-density fluid in the descending duodenum, suggestive of hemorrhagic fluid.

After successful placement ( Fig. 2 ) and suturing of a gastrogastric 20 × 10 mm lumen-apposing metal stent (LAMS), the excluded stomach was entered ( Video 1 ). An ulcer with a pigmented spot was found in the first portion of the duodenum ( Fig. 3 ), which was treated using bipolar cautery. Given the patient’s religious preference to avoid blood products, we then placed a 20 × 10 mm LAMS to tamponade the ulcer and allow healing ( Fig. 4 ). Both LAMS were removed 1 month later with complete ulcer healing ( Fig. 5 ).

**Fig. 2:**
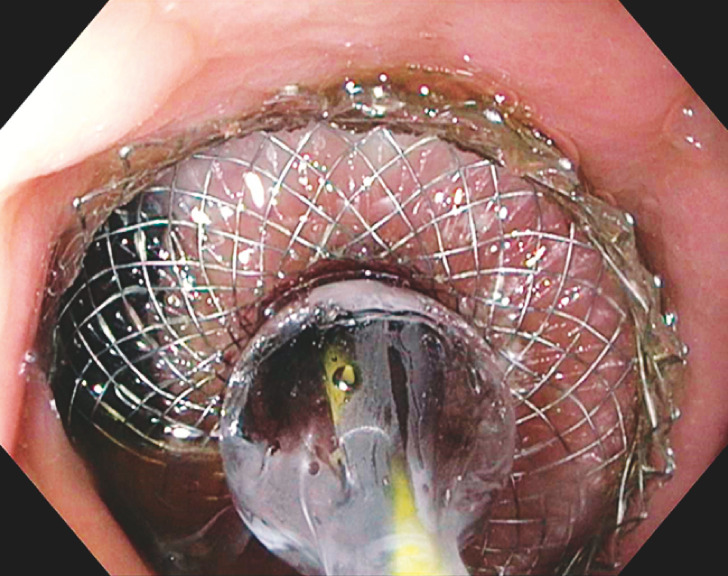
Balloon dilation of a gastrogastrostomy lumen-apposing metal stent.

**Fig. 3:**
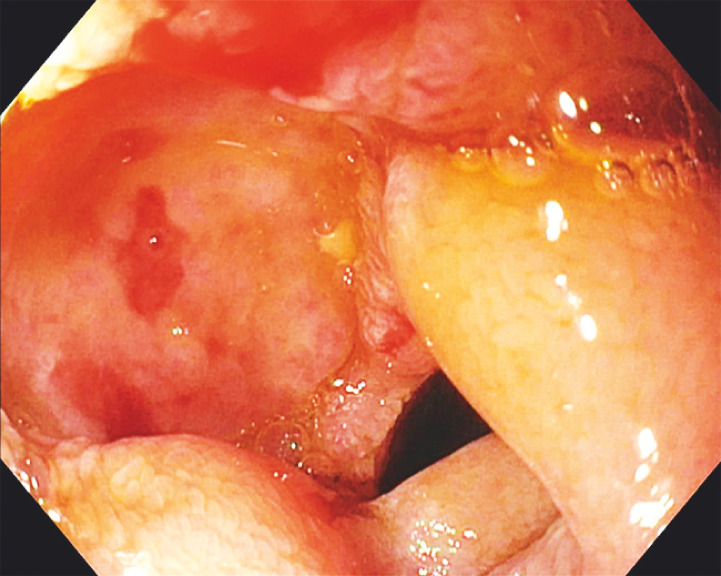
Pigmented spot within a large duodenal ulcer.

**Fig. 4:**
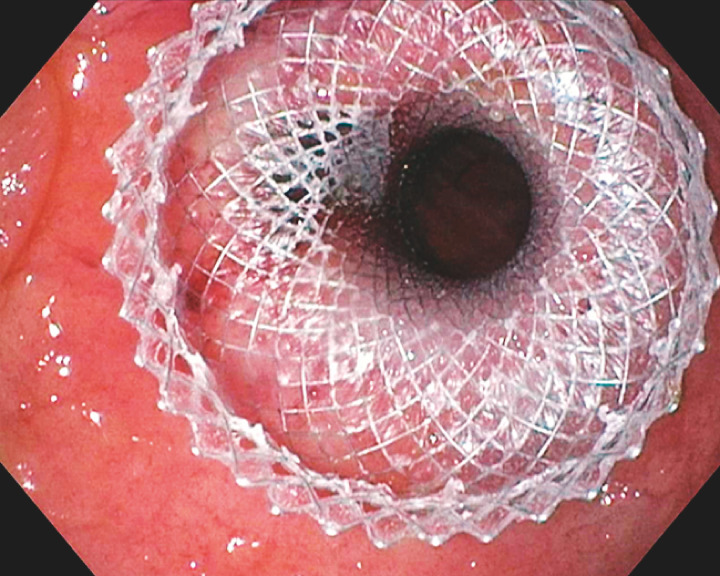
Placement of a 20-mm lumen-apposing metal stent to tamponade the duodenal ulcer.

**Fig. 5:**
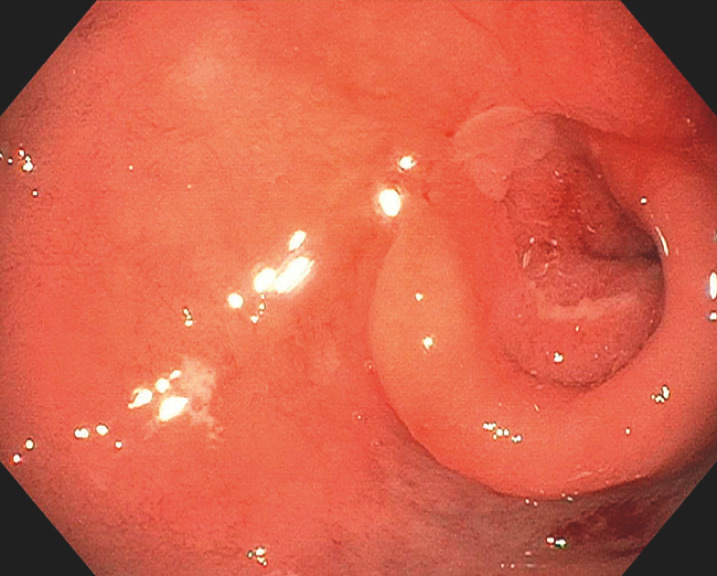
Resolution of the duodenal ulcer 1 month after index treatment.

Demonstration of endoscopic ultrasound-directed transgastric endoscopy for the treatment of upper gastrointestinal bleeding in a patient with Roux-en-Y gastric bypass.Video 1

The EDGE procedure allows access to the excluded stomach and duodenum in patients with RYGB anatomy 1 . We describe the use of single-session EDGE to treat gastrointestinal bleeding secondary to peptic ulcer disease. We also highlight how a luminal LAMS was utilized to create a tamponade effect, reducing the exposure to acidic and pancreaticobiliary contents and thus the risk of rebleeding, which was particularly important in this case, given the patient’s religious beliefs.

Endoscopy_UCTN_Code_TTT_1AS_2AB

## Notes

Endoscopy E-Videos https://eref.thieme.de/e-videos
*E-Videos* is an open access online section of the journal *Endoscopy* , reporting on interesting cases and new techniques in gastroenterological endoscopy. All papers include a high-quality video and are published with a Creative Commons CC-BY license. Endoscopy E-Videos qualify for HINARI discounts and waivers and eligibility is automatically checked during the submission process. We grant 100% waivers to articles whose corresponding authors are based in Group A countries and 50% waivers to those who are based in Group B countries as classified by Research4Life (see: https://www.research4life.org/access/eligibility/ ). This section has its own submission website at https://mc.manuscriptcentral.com/e-videos .
